# MS imaging of multicellular tumor spheroids and organoids as an emerging tool for personalized medicine and drug discovery

**DOI:** 10.1016/j.jbc.2021.101139

**Published:** 2021-08-28

**Authors:** Yijia Wang, Amanda B. Hummon

**Affiliations:** Department of Chemistry and Biochemistry, Comprehensive Cancer Center, The Ohio State University, Columbus, Ohio 43210, USA

**Keywords:** MS, imaging, cell culture, tumor therapy, pharmacology, BBB, blood–brain barrier, CTO, colon tumor organoid, DESI, desorption electrospray ionization, DHB, 2,5-dihydroxybenzoic acid, ESI, electrospray ionization, FOLFIRI, FOL: folinic acid/leucovorin, F: 5-fluorouracil (5-FU), and IRI: irinotecan, IRI, irinotecan, IS, internal standard, LAICP, laser ablation inductively coupled plasma, MCTS, multicellular tumor spheroid, ML, machine learning, MSI, MS imaging, mTHPP, 5,10,15,20-tetrakis(3-hydroxyphenyl)porphyrin, PBC, paper-based cell culture, PCA, principal component analysis, PDO, patient-derived organoid, qMSI, quantitative MSI, SIMS, secondary ion MS, TIC, total ion count

## Abstract

MS imaging (MSI) is a powerful tool in drug discovery because of its ability to interrogate a wide range of endogenous and exogenous molecules in a broad variety of samples. The impressive versatility of the approach, where almost any ionizable biomolecule can be analyzed, including peptides, proteins, lipids, carbohydrates, and nucleic acids, has been applied to numerous types of complex biological samples. While originally demonstrated with harvested organs from animal models and biopsies from humans, these models are time consuming and expensive, which makes it necessary to extend the approach to 3D cell culture systems. These systems, which include spheroid models, prepared from immortalized cell lines, and organoid cultures, grown from patient biopsies, can provide insight on the intersection of molecular information on a spatial scale. In particular, the investigation of drug compounds, their metabolism, and the subsequent distribution of their metabolites in 3D cell culture systems by MSI has been a promising area of study. This review summarizes the different ionization methods, sample preparation steps, and data analysis methods of MSI and focuses on several of the latest applications of MALDI-MSI for drug studies in spheroids and organoids. Finally, the application of this approach in patient-derived organoids to evaluate personalized medicine options is discussed.

MS has become a powerful and versatile method for the study of new drugs. Because of its ability to detect numerous molecular species at the same time, it has been employed in the clinical field, with uses ranging from drug discovery to personalized medicine (1). In its most common application, biological samples are homogenized and analyzed for their molecular composition using LC–electrospray ionization–tandem MS (LC–ESI–MS/MS). While powerful to discern the identity, and frequently also the quantity, of hundreds of biological molecules, this type of traditional MS analysis lacks information on spatial distribution, which is of great importance for heterogeneous biological samples. To also obtain spatial distribution, MS imaging (MSI) was developed to interrogate both the localization and concentration of molecules, and this approach has been employed in a growing number of medical studies (2).

MSI is a label-free method that is used to map the distribution of molecules in different biological samples (3). It was first employed in biological tissues by Caprioli *et al.* (4) in 1997 using MALDI-TOF MS to visualize proteins and peptides. Since that first study, the types of molecules imaged have been substantially expanded in scope and now include both endogenous (5) and exogenous (6) metabolites, lipids (7), and drugs (8). Compared with traditional clinical imaging methods requiring the use of indirect labels such as radiolabels or probes, MSI not only has high spatial resolution in low micrometer scale but also can simultaneously detect the spatial distribution of a drug and its resulting metabolites, showing great potential to study drug mechanisms and efficiency (9).

Various biological samples have been used to evaluate therapeutics by MSI, such as whole animal bodies (10), tissue samples (11), and 3D cell cultures (12). Tissue and animal samples have played an important role in MSI for decades, but they are relatively expensive and time consuming. Therefore, *in vitro* cell culture models are attractive alternatives.

2D cell culture is the conventional cell-based biological model traditionally used for initial drug testing. In 2D cell cultures, immortalized cells are grown as a monolayer or in suspension. This model system has many positive attributes; it is simple, fast to grow, easy to manipulate, and relatively cost effective. However, while the simplicity of the 2D cell culture model makes it convenient, the lack of complexity also means that it fails to mimic many aspects of the tumor microenvironment. 3D cell cultures, also known as multicellular tumor spheroids (MCTSs), are scaffold-free and micron-sized self-assembled aggregates of epithelial tumor cells. MCTSs were developed by Sutherland *et al.* (13) in 1971 at the University of Rochester, providing a useful *in vitro* model for assessment of the effects of drug treatment. These 3D cellular aggregates can be grown in multiple ways. The most common growth method, the liquid overlay approach, involves seeding cells in media in a concave agarose meniscus. Because of the concavity of the surface, the cells are forced into contact with each other and establish cell–cell connections forming an aggregate. The spheroids typically grow to ∼1 mm in diameter over 2 weeks. Other methods, such as the hanging drop approach, are designed to similarly force the cells into contact with each other in a concave growing space, so that they can form cell–cell connections.

MCTSs are a valuable model system for many reasons. They capture the advantages of 2D cell culture; simple, relatively fast, easy to manipulate, and lower cost than animal models. More importantly, they are a better mimic of the heterogeneity found in avascular microregions of tumors. In poorly vascularized tumors, radially symmetric chemical gradients develop because of poor diffusion within the cell mass (Fig. 1) (14). For example, oxygen concentrations vary substantially throughout the structure, with normal oxygen concentrations (normoxic) at the outer rim and low oxygen (hypoxic) conditions existing in the center (15). These chemical gradients result in radially symmetric cellular gradients. For example, cells proliferate with the higher concentrations of nutrients and oxygen in the outer layer, followed by quiescent cells in the middle layer and necrotic cells in the core region.

**Figure 1:**
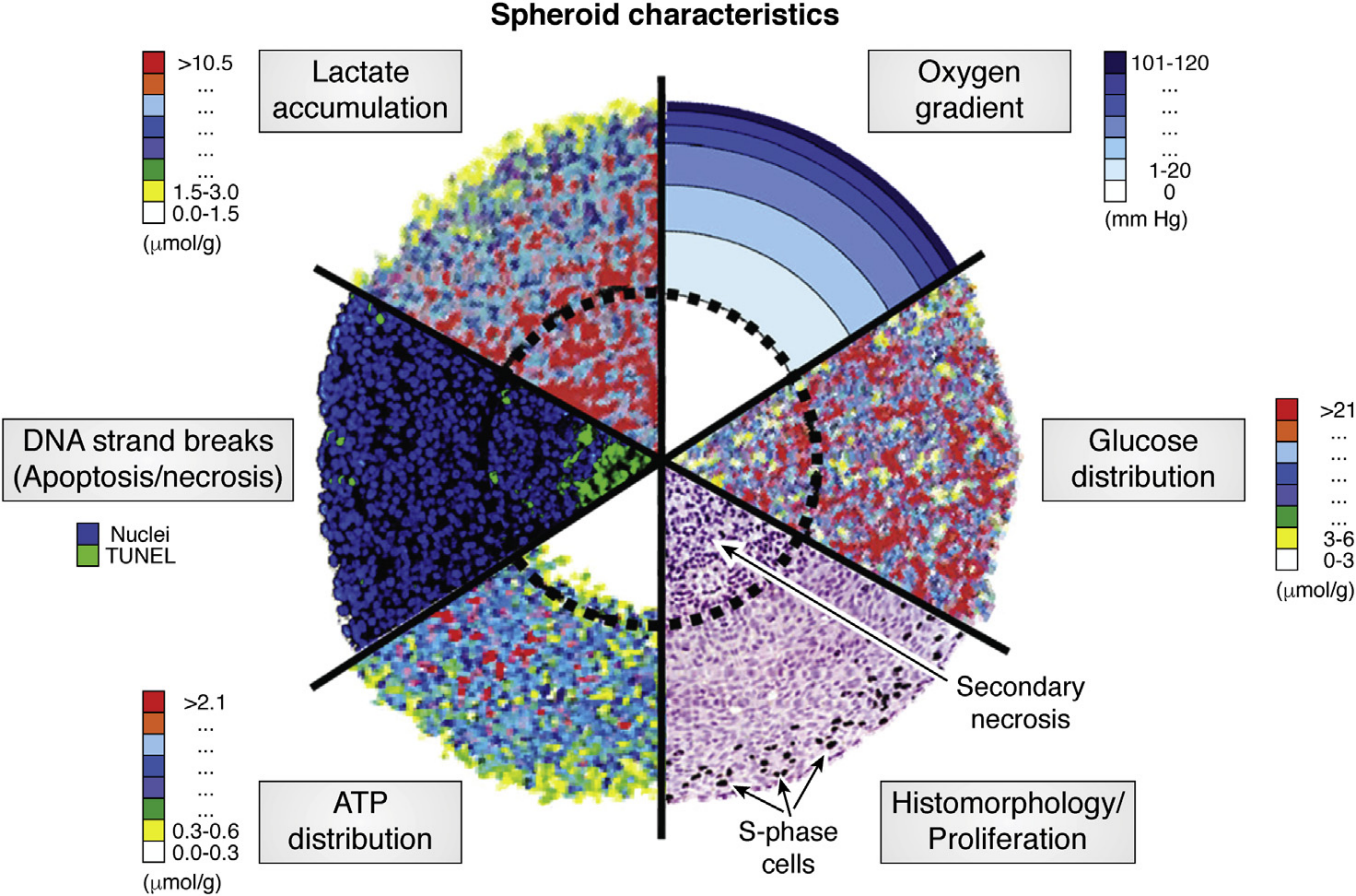
**Combination of analytical images of spheroid sections showing the concentric arrangement of cell proliferation, viability, and the chemical micromilieu.** This figure was reprinted with permission from Ref. (14). Copyright (2010) Elsevier B.V.

In 2011, Li and Hummon (16) were the first to analyze a MCTS system by MSI, successfully mapping several proteins in colon carcinoma HCT 116 spheroids. In the succeeding decade, there have been numerous reports of MSI applied to MCTS, both to explore the underlying biology and to evaluate the penetration and metabolism of new drug compounds (17, 18, 19, 20, 21). This review will focus primarily on the application of MSI to the MCTS model. We will also briefly introduce a novel model system, the patient-derived organoid (PDO), as the next-generation platform for these types of studies.

## Different ionization methods used for MSI

One of the most critical steps that influence the quality of a MS experiment is the ionization of the analytes of interest. Various ionization techniques have been employed in MSI studies, such as MALDI, desorption electrospray ionization (DESI) (22), secondary ion MS (SIMS) (23), laser ablation electrospray ionization (24), and laser ablation inductively coupled plasma (LAICP) (25). All these technologies have advantages and disadvantages.

In 1997, Caprioli *et al.* (4) first developed an MSI method using MALDI. With this soft ionization source, MALDI imaging is a powerful tool to detect large molecular weight compounds, such as peptides, proteins, and polymers, and was later expanded to image small molecules, such as drugs, metabolites, and lipids. In part, with its advantages of high tolerance of salts and buffers, fast analysis time, and high sensitivity (26), it has become the most popular technique for MSI. While MALDI is widely utilized, one challenge with the approach is the presence of matrix interference as there is typically a large signal corresponding to the *m/z* value of the matrix. This issue can be especially problematic in studies of lower molecular compounds, like drugs and metabolites. To avoid matrix interference, matrix-free desorption/ionization methods are of great importance. Predating the innovation of the soft ionization techniques like MALDI and ESI, SIMS was developed as one of the first matrix-free methodologies (27). In a SIMS experiment, a primary ion beam with high energy sputters a solid surface to produce secondary particles, and the secondary ions are detected to create a mass spectrum (23). This technique has excellent nanometer-scale spatial resolution and is most sensitive to analyze surface molecules including proteins and lipids (28). However, it has higher limits of detection compared with the other MSI techniques (29).

DESI is another matrix-free ionization method, first developed by Cooks *et al.* in 2004 (22). DESI is an ambient soft ionization technique. Ionization occurs when an electrically charged mist is directed at a surface a few millimeters away, causing desorption and ionization of analytes from the surface in the splashed droplets. A significant advantage of DESI is that it does not need high vacuum and allows analysis under atmospheric conditions, unlike MALDI and SIMS. Also, sample preparation for DESI analysis is considerably simpler than the other approaches, as no matrix or complicated sample preparation is needed (30). DESI is used to detect various molecules, such as lipids (31), metabolites (32), proteins (33), and nitroaromatic compounds (34).

LAICP is another ionization approach that includes a laser ablation process involving the interaction between the laser beam and biological samples and a postionization process that ablates material in a inductively coupled plasma (25). It is primarily used to study trace metal elements and isotopes and has the great advantages of both high sensitivity and high spatial resolution, typically ranging from 10 to 100 mm (35). As metal elements often play crucial and unique roles in biological systems, LAICP has the potential to interrogate these functions, for example, endogenous elements in cells like abundant metal isotopes (24) Mg, ^66^Zn, and ^63^Cu (36). LAICP-based MSI can also be employed to examine the distribution of metal-based anticancer drugs like cisplatin (37) and nanoparticles like gold (38) and iron oxide (39).

Laser ablation electrospray ionization is another novel ambient ionization method that was developed by Nemes and Vertes in 2007 (24). The ionization process includes two steps. In the laser ablation process, the target molecules containing hydroxide groups absorb energy from the laser and evaporate to ablate with a small portion of the sample into the gas phase to generate the analyte species (40). Then the neutral analytes are ionized by an ESI source above the samples (41). By combining laser ablation from a midinfrared laser with a secondary ESI process, it allows direct analysis of biological samples with little sample preparation and is used to perform MSI analysis of various molecules ranging from small molecules such as lipids and metabolites (42, 43) to larger molecules such as proteins (44).

There are also other novel methods that have been developed for MSI analysis of small samples such as MCTS. For example, the single-probe analysis, with its small sampling probe size (<10 μm), is ideal for the MSI of MCTS, which benefit from the spatial resolution (45). While all the ionization methods mentioned previously have been used for imaging analyses, for brevity, the remainder of this article will only focus on MALDI-MSI studies, as it is the most widely used ionization approach.

Different aspects will be covered, including sample preparation methods, data analysis methods, and quantification studies. Each step is critical to MALDI-MSI studies. To begin with, sample preparation is the first step in MALDI-MSI analysis and is crucial because appropriate sample preparation ensures the maintenance of the spatial distribution and abundance of molecules of interest. Subtle variation in the sample preparation may significantly affect the signal intensities, ion generation, abundance, and localization in MSI analysis. Data analysis is the last step in MALDI-MSI and used to visualize the spectra and extract information from the large dataset. With the instrumentation developments and method optimization in sample preparation and data analysis, MALDI-MSI has been expanded from visualizing only one large biomolecule at a time to monitoring several compound classes and more complex systems. Also, better sensitivity and spatial resolution has been achieved. Although quantifying different molecules in small samples remains challenging because of limited target molecules, the continued improvements in MSI have shown great potential to achieve quantitative imaging on spheroids and organoids. This review is aimed at providing a brief tutorial for readers interested in MALDI-MSI studies aiming to get started in this field. We will also offer examples of application of MALDI-MSI on 3D cell culture model.

## Methods for sample preparation

**Figure 2:**
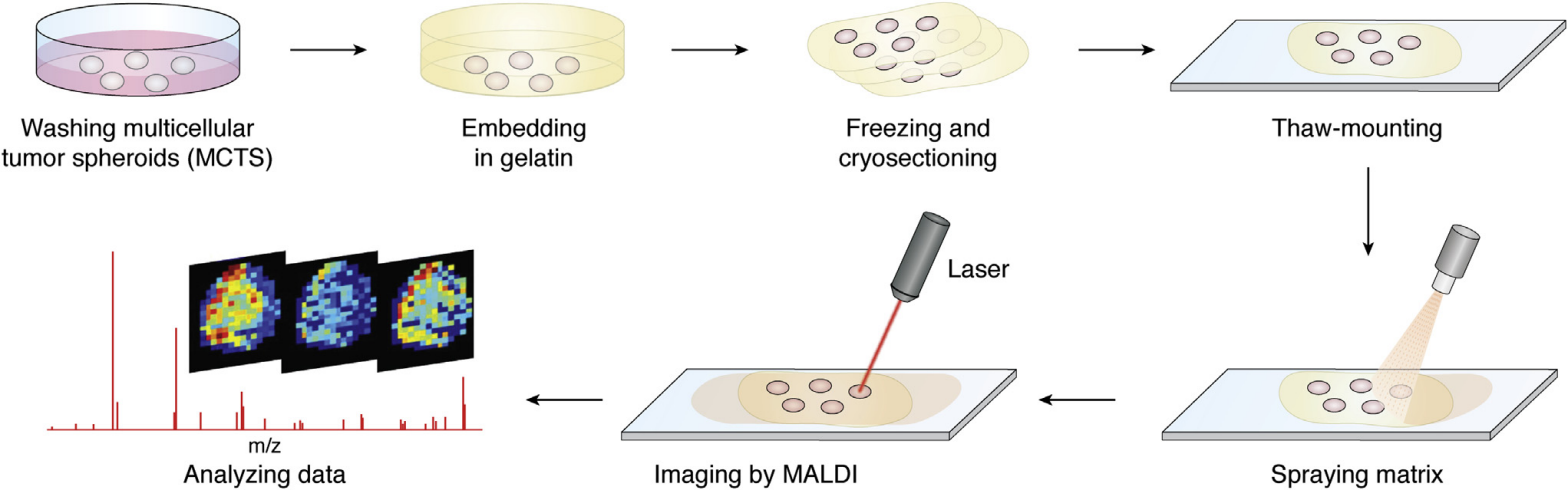
**General workflow of MALDI-MSI on MCTS.** MCTSs are collected when they grow to a stable size. (For HCT 116 in culture for 6000 initial cells, it is about 1 mm in diameter at day 14–20. (16)) The harvested MCTSs are washed with PBS to remove the cell culture media and then are embedded in gelatin. The samples are stored in −80 °C and cryosectioned to 12-μm thick slices. The sections are thaw mounted onto ITO slides. A matrix nebulizer can be used to spray the matrix solution onto the slides homogeneously, and MALDI-MSI can be performed. ITO, indium–tin oxide; MCTS, multicellular tumor spheroid; MSI, MS imaging.

The sample preparation process is vital for MALDI-MSI studies as it helps to maintain both the distribution and concentration of molecules of interest in the samples for analysis (46). General steps needed for MALDI-MSI sample preparation include cryosectioning, thaw-mounting sample slices onto indium–tin oxide sides, and matrix spraying (Fig. 2).

To perform MSI experiments, biological samples are cryosectioned prior to analysis. Typically, samples are frozen and then cryosectioned into 10- to 15-μm slices. For large samples, like intact animals or larger organs, the samples can be manipulated by hand to enable easy sectioning. For smaller samples, for example, 1-mm diameter spheroids or smaller organoids, it is helpful to embed the samples in a solid matrix that can be manipulated by hand. Typically, gelatin is used for embedding samples as it is readily available and does not produce a background signal in the MALDI analysis (47, 48). One disadvantage of gelatin is that it has limited transparency when frozen, making it hard to view the spheroids during sectioning. To solve this problem, ice-coated spheroids can be placed onto carboxymethyl cellulose for cryosectioning (49). The sectioned samples are then thaw mounted on glass slides by carefully flattening, so that they adhere onto the slides at room temperature. During the sampling process, the natural state of the sample can become altered because of folds, cracks, tears, or delocalization of the molecules in the tissue sections. These slides are typically glass slides coated with indium–tin oxide. In a MALDI experiment, a matrix is applied to the surface of sample sections in a homogeneous layer to cocrystallize with the analytes. There are numerous matrices available, and the choice of matrix is tailored to optimize ionization of the analyte of interest during the experimental design. As the sample is then irradiated with a UV laser, the matrix absorbs the energy and promotes the desorption and ionization of the molecules of interest in the sample.

**Figure 3:**
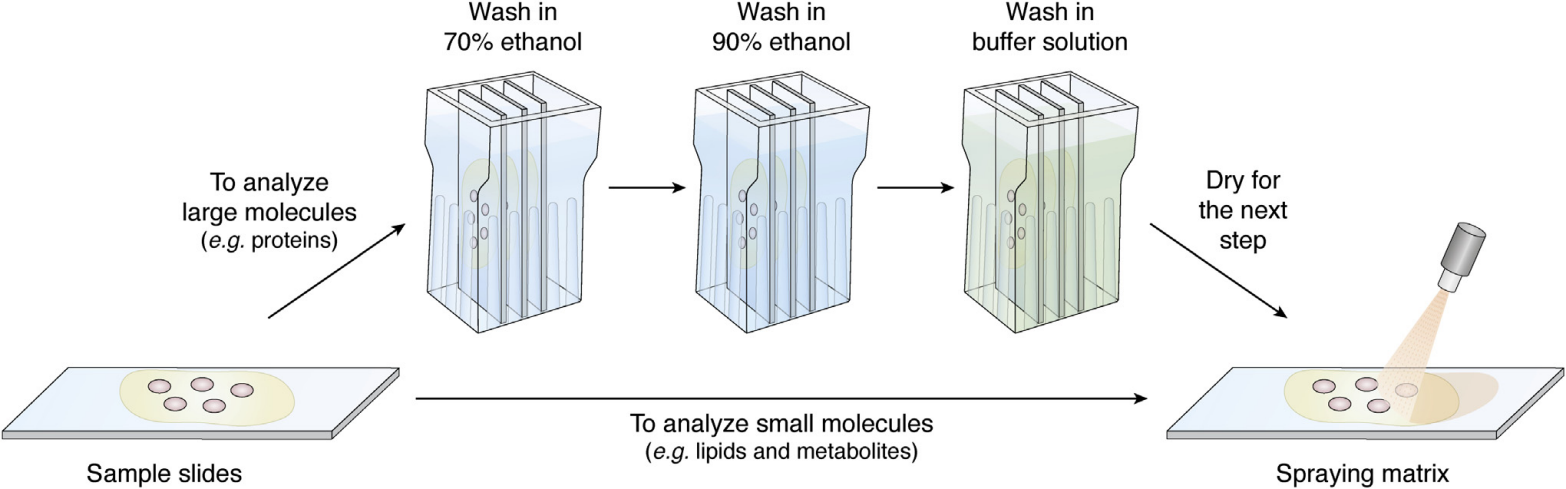
**Workflow of the washing steps for proteins MSI before matrix desorption.** Example taken from Ref. (56), to improve the signal of proteins, tissue samples were washed in 70% EtOH first, followed by 90% EtOH and buffer solution at last. MSI, MS imaging.

Based on the chemical differences of the analytes of interest, different steps in the method can be optimized, such as washing the slides, changing the matrix, or adding a derivatization reaction, to improve detection. Depending on the condition of the sample and analyte of interest, washing the slides prior to matrix spraying can be beneficial. Washing the sections should be carefully evaluated, as it can cause delocalization of the analyte of interest, especially small molecules. But in many cases, washing can enhance ionization and detection of the analyte, as is the case with high salt concentration samples. Usually, a washing step is necessary for analyzing large molecules such as proteins; when detecting small molecules, such as metabolites or lipids, this step can be skipped (50) (Fig. 3). Sometimes, washing steps can also benefit analysis of small molecules. Removing the salt can be particularly beneficial when the target drug molecule is insoluble. Washing with an ammonium acetate buffer solution will adjust the pH value while removing physiological salts and other soluble small molecules but still maintaining the location and concentration of the drug on the sample sections (51). In one study by the Li group, by serially washing with ammonium acetate solution, incubating samples with trifluoroacetic acid vapor, and washing with *n*-hexane, the pretreatment of MSI samples was optimized, enabling removal of not only salts but also some fat-soluble lipids to minimize the interference of those molecules in MALDI-MSI (52). To improve signal intensity of small-molecule metabolites (*m/z* <500) using MALDI-MSI, the group by Zhao *et al.* (53) found that cold chloroform could be used to wash the sections. With regard to the analysis of larger molecules such as proteins, an organic washing step is favored to remove endogenous salts and lipids and has been shown to stabilize the proteins (54, 55). Extra washes with a buffer solution may also benefit this process. For example, the laboratory of Chaurand *et al.* (56) determined that washing the sample serially with alcohol followed by an aqueous-based buffer made of ammonium formate increased the total ion count (TIC) of proteins ionized from the sample 4-fold. All these examples indicate that a washing step can increase the sensitivity and selectivity of an MSI study, when applied appropriately with suitable solvents and sufficient time.

Homogeneous matrix spraying is crucial in MALDI-MSI studies to achieve high-resolution images; as a result, approaches to apply the matrix are of great importance. The laboratory by Li *et al.* (57) conducted a comparative study of several major matrix application methods to analyze small molecules (molecular weight <1000 Da) including manual application, using an airbrush or an automatic sprayer, and the sublimation technique (58). Of the methods evaluated, they determined that the optimized automatic sprayer application was most effective at increasing the spatial resolution for small molecule detection.

There are various commercial matrices that are popular, such as 2,5-dihydroxybenzoic acid (DHB) and α-cyano-4-hydroxycinnamic acid for metabolite and peptide detection in positive mode (59), sinapinic acid for protein detection in positive ion mode (60) and 1,5-diaminonaphthalene (61), and 9-aminoacridine (62) for metabolite detection in negative ion mode. Several commonly used matrix and their properties are listed in Table 1. The combinations of different matrices may also improve matrix efficiency. For example, the binary use of DHB and 2-hydroxy-5-methoxybenzoic acid (super DHB) was shown to increase the number of carbohydrates and glycoproteins detected and provides greater signal intensity and improved reproducibility (63). Also, combining α-cyano-4-hydroxycinnamic acid and DHB can enhance ionization and detection of peptides (64). While most MALDI matrices are small organic acids, other classes of molecules, notably carbon allotropes and metals, have also shown utility. Graphene (65) and nanoparticles such as silver (66), gold (67), and TiO_2_ (68), have been used successfully as novel matrices to detect a variety of low-mass molecules (molecular mass <500 Da), such as steroids, amino acids, polyamines, anticancer drugs, nucleosides, and nucleotides (69).

**Table 1:** Commonly used UV MALDI matrices and their properties

Compound	Abbreviation	Wavelength (nm)	Analyte chemical	Applications
α-cyano-4-hydroxycinnamic acid	CHCA or HCCA	337, 355	Peptides, lipids, and nucleotides	Ref. (56)
2,5-Dihydroxy benzoic acid	DHB	337, 355, 266	Small peptides, nucleotides, oligonucleotides, oligosaccharides	Ref. (112)
1,5-Diaminonaphthalene	DAN	355	Oligonucleotides, peptides	Ref. (113)
9-Aminoacridine	9AA	337	Lipids, metabolites	Ref. (61)
3,5-Dimethoxy-4-hydroxycinnamic acid	SA	337, 355, 266	Large peptides, proteins, lipids	Ref. (114)

Even with optimized washing and matrix application, some classes of molecules can be difficult to detect, among which are some small molecules like metabolites and drugs. Although these molecules could be detected using other ionization methods, the limited access to these instruments and the high cost for sample analysis remain problematic. To better target these molecules using MALDI, chemical reactions can be performed to alter the physicochemical properties on the sample to improve subsequent ionization in MALDI-MSI. One type of chemical reaction is on-tissue derivatization. Without modification, platinum shows modest ionization by MALDI. Diethyldithiocarbamate was used to react with the Pt atoms in the drugs oxaliplatin, cisplatin, and carboplatin. In the reaction, ionizable dimer and trimer complexes of Pt with diethyldithiocarbamate were generated that showed three orders of magnitude increase in ionizability by MALDI-MSI as compared with the unmodified drugs. A distinct metabolite of the Pt drugs was also detectable, resulting in the simultaneous mapping of these Pt drugs and the metabolite in MCTS with MALDI-MSI. This approach could be extended to other metal-based drugs. Also, as cancer cells frequently have higher concentration of many metal ions compared with normal cells (70), this approach has great potential to map other metal ions in cells.

Another type of reaction is on-tissue reduction, which is used to map large molecules such as antibodies by breaking their disulfide bonds. Using DTT to break the disulfide bonds in the 180 kDa monoclonal antibody cetuximab, the two light chains (25 kDa) were produced. While the 180 kDa precursor could not be detected, the smaller 25 kDa light chains were ionized by MALDI-MSI, enabling localization in MCTS derived from two colon cancer cell lines (50).

## Data analysis, software, and application of machine learning

Over the last few years, MS instrumentation has advanced enormously. The development of instruments with improved spatial and mass resolution has resulted in an explosion in the size, complexity, and dimensionality of MSI datasets. With these complex datasets, it is imperative to have robust automated processing approaches (71).

**Figure 4:**
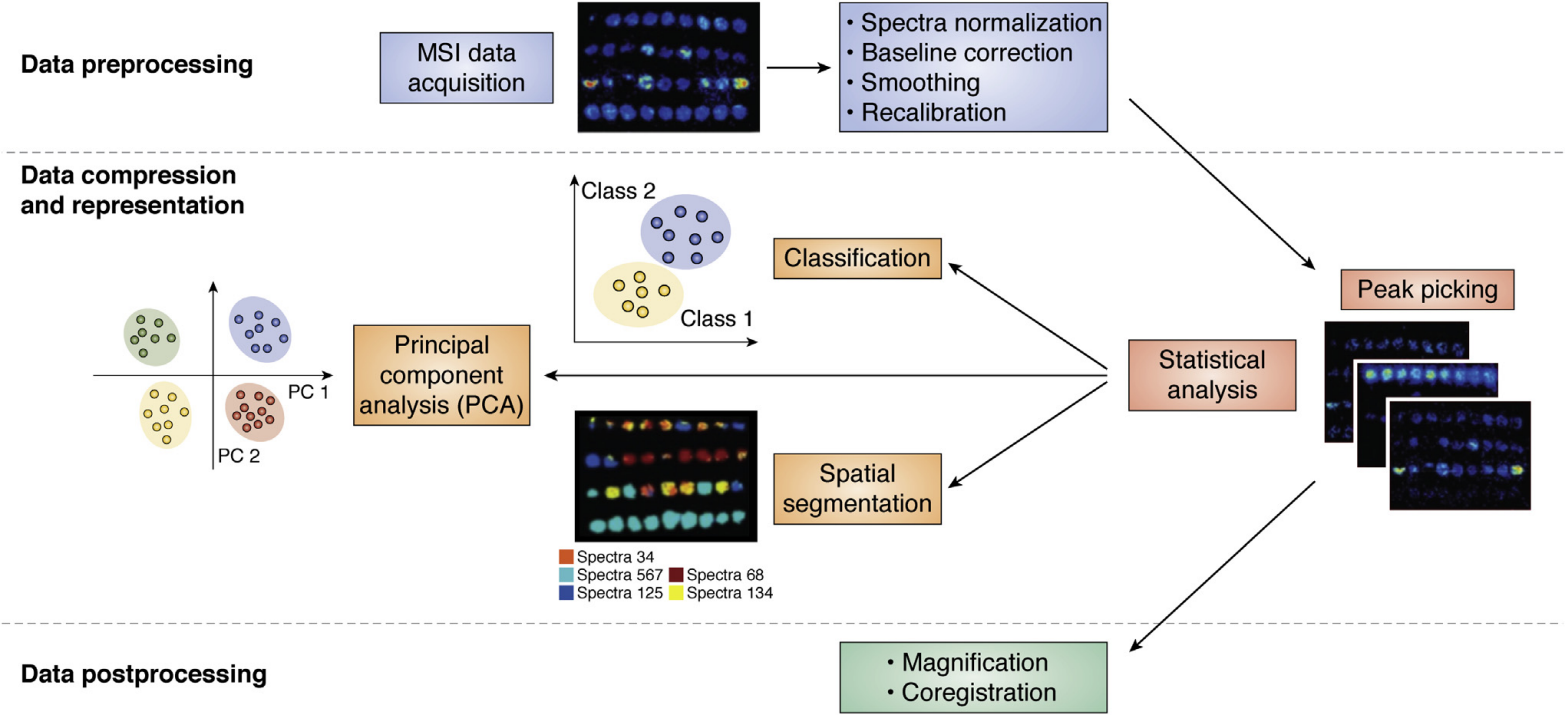
**Data processing and data analysis of MSI data.** MSI data are preprocessed after acquisition. This process includes several steps including spectra normalization, baseline correction, smoothing, and recalibration. Next, MSI data will undergo compression and representation, which reduces the computational load. Then data analysis could be completed, including peak picking and other statistical analyses, such as classification, principal component analysis (PCA), spatial segmentation, and others. Machine learning is a popular method used in these analyses. Postprocessing includes magnification and coregistration of the images. Adapted with permission from Ref. (29). Copyright (2018) American Chemical Society. MSI, MS imaging.

There are several steps to extracting meaningful information from MSI datasets. MSI data analysis requires conversion from abstract raw data into a visualized form based on the *m/z* values of the molecules of interest (72). Data processing usually has the following steps: data preprocessing, data compression and representation, and postprocessing (Fig. 4) (73). The preprocessing step includes spectra normalization, baseline correction, smoothing, and recalibration. These steps help to reduce experimental variance and extract meaningful information based on different research purposes so that they ensure the accuracy and efficiency of data analysis. There are several commonly used normalization methods, such as TIC, median normalization, or normalization to a manually selected *m/z* peak (74). Among all these methods, TIC is the most commonly used one, where all mass spectra are divided by their TIC. This approach works well in homogenous samples (75). Median normalization has been used for label-free proteomics studies (76). Normalization to a manually selected *m/z* peak is usually achieved with an internal standard (IS) in the sample, which has the advantage of reducing the error caused by sample inhomogeneity and pixel-to-pixel variability.

Data compression and representation is a process that reduces the computational load, which includes peak analysis and several statistical analyses, such as principal component analysis (PCA), classification, and spatial segmentation. Peak analysis can be completed by manually selecting the *m/z* of molecules of interest to visualize the distribution. PCA is capable of providing an overview of complex data by decreasing the dimensionality based on uncorrelated features called principal components. It can be used to reveal relations among samples or remove unrelated noise based on the clustering in the PCA plot (77).

Classification is used to distinguish different sample types. This can be achieved by partial least-squares regression, which is similar to PCA but has different ways of generating variances. Spatial segmentation is used to identify colocalized *m/z* by reconstructing images based on spectra similarity and separating different spectra into regions (78). Postprocessing includes magnification and coregistration of the images, allowing investigators to examine the imaging data for further research.

The size of datasets increases with improvements in sample size, spatial resolution, and mass resolution, which make the data load up to several gigabytes for most experiments (79). Because of the large size of MSI datasets, software used to perform data analysis should be able to process high-throughput data. There are commercial software packages available for MSI data analysis, such as FlexImaging (Bruker Daltonics), ImageJ (National Institutes of Health and the Laboratory for Optical and Computational Instrumentation [University of Wisconsin]), MSiReader (North Carolina State University), and SCiLS Lab (Bruker). The various software for statistical analysis can robustly provide reproducible results for fundamental research purposes (80).

**Figure 5:**
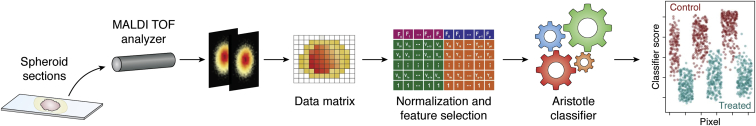
**Supervised machine learning process to analytes MSI data of the spheroids** After collecting MSI data in a matrix containing “samples” and “features,” cardinal is used to perform machine learning on these features to classify different samples into “control” or “treated.” Reprinted with permission from Ref. (82). Copyright (2020) American Chemical Society. MSI, MS imaging.

To complete statistical analysis of the large amounts of MSI data more efficiently and accurately, machine learning (ML) is starting to play an important role in examining the data and making predictions for unknown datasets (29). ML includes two types, supervised and unsupervised ML algorithms (81). Supervised ML methods learn the images on the differences among datasets and are proficient at discriminating groups (Fig. 5). Therefore, they are most commonly used for classification studies. For example, Hua *et al.* (82) developed an open-source supervised ML approach and analyzed MSI data of small molecules in sets of MCTS, successfully classifying drug-treated and untreated spheroids. Compared with supervised ML algorithms, unsupervised ML methods do not focus on categories of different datasets but try to discover trends, correlations, and associations within MSI data. Thus, unsupervised learning could be used for PCA and nonnegative matrix factorization and clustering or segmentation (83). Combining supervised and unsupervised ML methods together could provide a more complete understanding of molecular distribution and benefit drug studies. Tian *et al.* employed both supervised and unsupervised ML methods to analyze MS images of metabolites in drug-treated MCTS. Unsupervised ML was completed by the use of Clustering for Large Applications to directly classify the MSI data into three regions according to the similarities of MS profiles, the inner spheroid, the outer spheroid, and the background. Supervised ML was followed based on selected training data from multivariate curve resolution alternating least square algorithms, clustering the data into three regions similar to unsupervised ML results. The consistent results ensured the accuracy of the classification. Furthermore, the comparison of relative intensities of metabolites from unsupervised and supervised ML gave a more accurate trend of the changes in the metabolites, reducing the error caused by the variances in relative ion intensities from different methods. It was also found that irinotecan could significantly influence the distribution of different categories of metabolites within distinct spatial areas of MCTS (84).

Two software platforms are utilized most often for MSI ML analyses, Matlab (http://uk.mathworks.com/products/matlab) (85) and R (https://www.r-project.org/) (86). These options are more difficult to use compared with commercial software because they require computational knowledge in programming, but they have the advantage of more features and flexibility through their customization (80).

The development of data analysis methods has vastly improved the efficiency and accuracy for MALDI-MSI studies, which has made MSI a more user-friendly method. This contributes to maturing the technique for bioanalysis and pharmaceutical discovery.

## Quantification studies

Quantification of drugs and metabolites in a spatially defined manner is valuable for drug discovery. Therefore, it has become an important component in many MSI studies (87). Quantification by MSI is achievable but adds to the complexity of the experiment. This challenge is especially evident in MALDI-based studies, as complicating factors, like sample surface heterogeneity, matrix coating heterogeneity, stability of analytes, inefficient analyte extraction, and ionization suppression effects, affect the MALDI signal (88). Therefore, more considerations should be taken to achieve MALDI quantitative MSI (qMSI), including sample preparation protocols, the influence of sensitivity by matrix selection, construction of calibration curves, signal normalization, and visualization of MSI data (74). Although difficult, both absolute (89) and relative (90) quantitative studies have been completed using MALDI-MSI on tissue samples, mostly evaluating the concentration of small molecules (91), with relative quantification studying concentration differences from experimental conditions. Absolute quantification experiments typically calculate the specific molecular concentration directly.

qMSI has been performed in 3D cell culture systems. For example, using an IS method and developing matrix-matched standards, quantitative bioimaging was achieved to map Pt and Pd elements in tumor spheroids by LAICP-MS. This method was used to evaluate the penetration of Pt- and Pd-based drugs into the TFK-1 spheroids, such as cisplatin, and the Pd-tagged photosensitizer 5,10,15,20-tetrakis(3-hydroxyphenyl)porphyrin (mTHPP). After 6-h incubation of Pt(II)acetylacetonate, the entire spheroid showed an average concentration of Pt at 1.1 μg/g, with those values primarily quantified in the quiescent and necrotic cell layer. A concentration of up to 6.9 μg/g Pt was assessed in the outer cellular layer. After an incubation time of 24 h, an average concentration of 2.0 μg/g Pt was detected with a maximum concentration of 37.2 μg/g Pt in the proliferating cell zone. The Pd-based mTHPP was successfully quantified with the average concentration of 25.7 μg/g Pd after 24-h treatment and 100.6 μg/g Pd after 48-h treatment. Comparison of the different localization of the two analytes showed that though both of them had high enrichment, cisplatin fully penetrated the spheroid after 24 h, because of its hydrophilic character, whereas mTHPP-Pd accumulates more in the outer layer of the spheroid because of its hydrophobicity. While the precise quantification of compounds is an asset, another advantage of this approach is the spatial resolution, which can approach 5 μm (92).

MALDI-qMSI in individual spheroids remains challenging because of the limited sample size. There is one study quantifying irinotecan on paper-based cell cultures (PBCs) with 2.85-mm diameter zones. Irinotecan with a gradient concentration (0.05–50 μM) was spotted onto PBCs containing only Matrigel. With irinotecan-d10 sprayed as an IS, a calibration curve was obtained, showing the possibility of quantifying irinotecan on PBCs at this range. Spatial segmentation of paper scaffold imaging data was obtained to better distinguish the cell zones, drug zones, and Matrigel zones (93).

While these advances move the field closer, no MALDI-qMSI has yet been achieved on individual MCTS because of their smaller sample size, less than 1 mm in diameter. Although LC–MS/MS remains an accurate method to perform quantitative studies on MCTS, it lacks the information of localization and consumes more time for sample preparation and analysis. qMSI has shown great potential as a comparable method. Further efforts will be needed to develop the qMSI methods for the application to single MCTS (94).

## Application of MALDI-MSI in drug studies

The identification of appropriate pharmacological targets is critical to drug discovery. The dosing of drugs usually results in significant changes in biochemical processes. By monitoring and comparing the biomarkers in dosed and undosed samples or normal and diseased samples, we can have a better understanding of the possible causes of diseases and how different drugs response in the treatment (95).

MALDI-MSI is capable of analyzing multiple different molecules at the same time. This property is especially beneficial in studies characterizing new therapeutics, when it is necessary to evaluate both the drug and its metabolites. In many microscopy-based approaches, where a chemical or a fluorescent tag is attached to the species of interest, it can be difficult to distinguish among the various related molecules. With MS, if the masses are distinct, it is simple to examine them individually. Even in cases where the masses are isobaric, newer separation approaches, like ion mobility, can provide the ability to discriminate among them by their shape (96).

In these MALDI-MSI studies of drugs and their metabolites, it is common to evaluate the time-dependent penetration and conversion of the drug compound. It is important to study the penetration of drugs because understanding of the drug distribution is one of the major goals for pharmaceutical development, but there are limited studies focused on the delivery of anticancer drugs to the tumor cells (97). Therefore, studying the way anticancer drugs penetrate solid tumors can help to evaluate the drug efficiency, and the investigation of their pharmacological properties could help to determine the minimum effective concentration of a pharmaceutical (20). The first example of this approach was an examination of irinotecan in HCT 116 colon carcinoma spheroids. MCTSs were treated with the IC_50_ of irinotecan (20.6 μM) for different amounts of time and then evaluated by MALDI-MSI. The penetration of irinotecan into spheroids increased over time, with complete penetration into the 1-mm diameter spheroids at 24 h. For the 72-h drug-treated spheroids, not only the drug but also its three metabolites, an active metabolite SN-38 (*m/z* 393), an inactive metabolite SN-38 glucuronide (*m/z* 569), and a decarboxylation metabolite (*m/z* 543), were imaged successfully (17).

**Figure 6:**
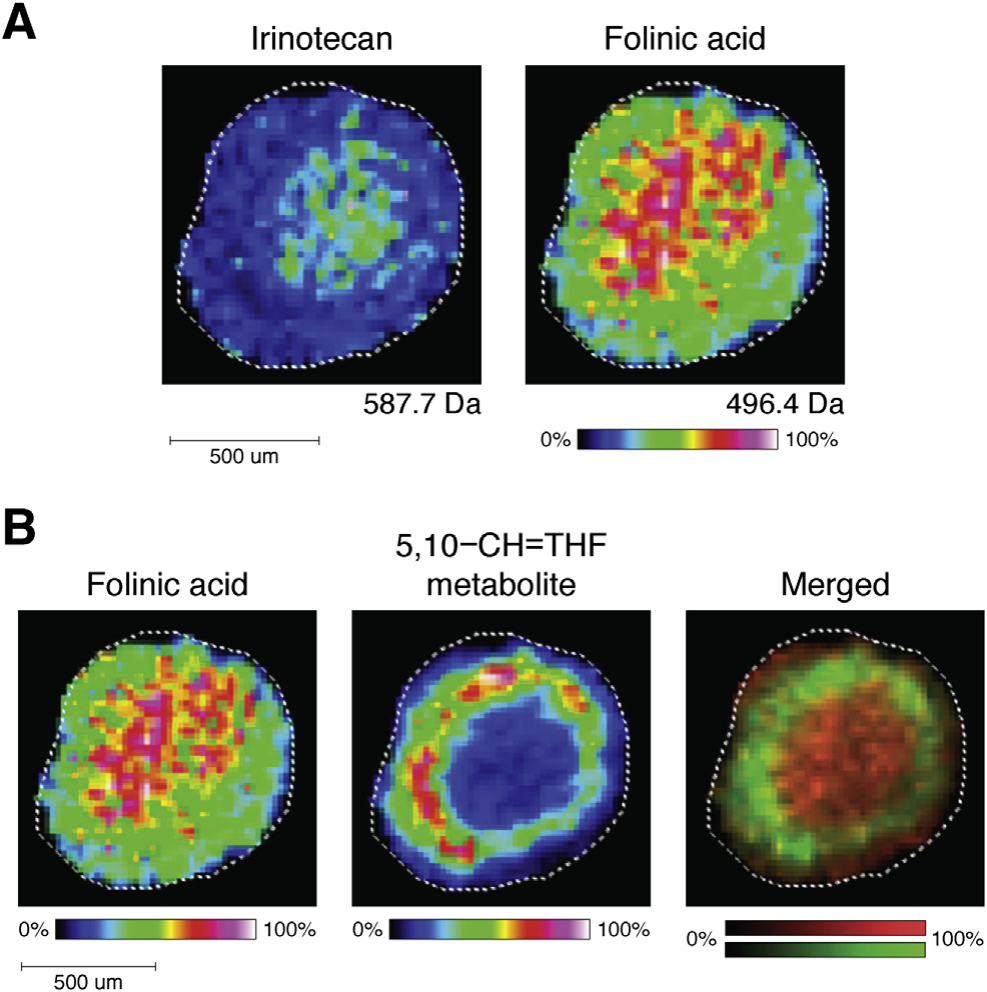
**Image of FOLFIRI-treated HCT 116 spheroids.***A*, localization of irinotecan and folinic acid within 24-h treatment. *B*, a metabolite of folinic acid was detected in the proliferating layer of the spheroids. Reprinted with permission from Ref. (115). Copyright (2018) American Chemical Society. FOLFIRI, FOL: folinic acid/leucovorin; F: 5-fluorouracil (5-FU); and IRI: irinotecan.

In a follow-up study, MALDI-MSI was used to analyze MCTS treated with FOLFIRI (FOL: folinic acid/leucovorin; F: 5-fluorouracil [5-FU]; and IRI: irinotecan), a combination chemotherapy regimen. Chemotherapies are often administered in combinations to take advantage of multiple mechanisms of action. FOLFIRI is a common treatment for advanced colorectal cancer (98). HCT 116 spheroids were dosed for 24 and 48 h with FOLFIRI and then evaluated by MALDI-MSI. In the 24-treated spheroids, folinic acid and irinotecan were detected with higher concentrations in the necrotic center as compared with periphery of the spheroids. A metabolite of folinic acid, 5,10-CH-THF (*m/z* 456.4), was mapped to the quiescent and proliferative layers of the MCTS. These results correspond with the anticipated drug metabolism as the outer proliferating layers are more active in converting folinic acid. 5-FU was challenging to be detected by MALDI-MSI because of its low molecular weight (130 g/mol), and thus, it was extracted and detected using LC–MS. This indicates the challenges of detecting combination regimens using MALDI-MSI, as a result of the different ionization efficiency. Although 5-FU was not imaged directly, it was the first time that the penetration of folinic acid and irinotecan into dynamically dosed MCTS was examined simultaneously. This study helped to expand the application of MALDI-MSI from one drug to a chemotherapeutic combination regimen (Fig. 6).

MALDI-MSI is also applicable to map drugs and metabolites with different drug delivery configurations, such as liposomes. A liposomal drug carrier system was synthesized with lipids containing head groups of phosphatidylcholine, phosphatidylethanolamine, and cholesterol to help encapsulate and transport doxorubicin to MCTS. The system was evaluated by visualizing drug penetration using MALDI-MSI. Both free and liposomal doxorubicin were mapped at different time points within 72 h at a final concentration of 30 μM doxorubicin. It was determined that the drug could fully penetrate the spheroids after 12-h treatment in all the samples, indicating that the liposomes do not significantly influence the penetration rate for doxorubicin. Also, at the 72-h time point, three metabolites of doxorubicin were imaged in both the free and liposomal doxorubicin-treated MCTS, with similar distribution patterns in both samples (19).

**Figure 7:**
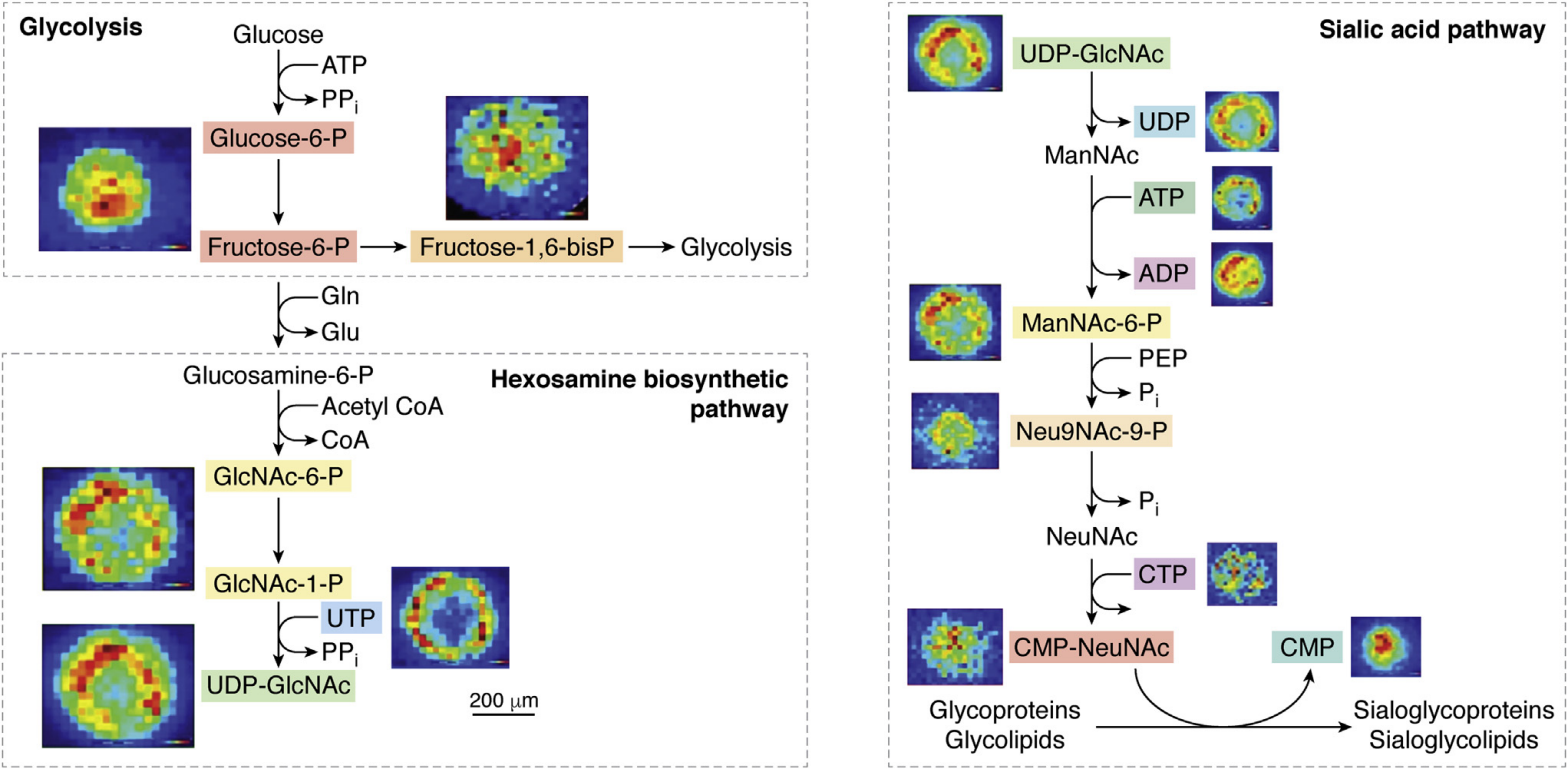
**Mapping of endogenous metabolites in MCF-7 breast cancer spheroids by MALDI-MSI.** Elemental formula of the metabolites assigned can be mapped onto the hexosamine biosynthetic pathway (HBP). *N*-acetyl neuraminic acid (sialic acid) is formed by the end product of the HBP. Adapted with permission from Ref. (18). Copyright (2019) American Chemical Society. CMP, cytidine monophosphate; CMP-NeuAc, cytidine monophosphate *N*-acetylneuraminic acid; GlcNAc-1-P, *N*-acetyl glucosamine-1-phosphate; ManNAc-6-P, *N*-acetylmannosamine-6-phosphate; MSI, MS imaging; NeuNAc, *N*-acetylneuraminic acid; Neu9NAc-9-P, *N*-acetylneuraminic acid-9-phosphate; UDP, uridine diphosphate; UDP-GlcNAc, uridine diphosphate-*N*-acetyl glucosamine; UMP, uridine monophosphate; UTP, uridine triphosphate.

High-resolution MALDI-MSI can also be used to study cellular metabolism pathways. Endogenous metabolites were evaluated in breast cancer MCF-7 spheroids using a MALDI mass spectrometer with a cyclotron FT-IR mass analyzer. Several endogenous metabolites were detected and selected as biomarkers to study the hypoxic and oxidative stress in the MCTS. High-energy uridine phosphates and high-energy cytidine phosphates, metabolites that represent different redox and energy components, were identified and mapped to distinct regions of the spheroids, matching the oxygen gradient within the MCTS. Comparing the localization of these metabolites back to the distinct pathways provides a better understanding of the microenvironment and the biochemical equilibria in different regions of the spheroids (Fig. 7) (18).

## MSI on organoids

Although the spatial heterogeneity of MCTS enables them to reproduce some aspects of tumor biology, their simple structure fails to mimic the morphological, phenotypic, and genetic heterogeneity of the complex tumor microenvironment in tissues. In particular, as most spheroids are grown using a single monoclonal epithelial cell line, they do not recapitulate the cellular complexity of *in vivo* tumors. Another complication is the fact that the formation of MCTS is dependent on cell adhesion properties (99). As a result, not all carcinoma cell lines will form spheroids, which can limit the ability to model different diseases and systems in preclinical research and has led researchers to develop more complicated and realistic 3D cellular model systems.

Organoids are self-organizing “miniorgans” at microscopic level (100), grown directly from biopsies. Both human patient and mouse-derived organoids have been developed (101, 102). Organoids are generated from progenitor cells, which are derived from stem cells. As the cells have the ability to reprogram and redifferentiate, organoids can mimic the complex heterogeneous microenvironment of their parent tissues (103). Therefore, the development of a patient-specific organoid model combined with MSI analysis offers great potential in therapeutic analysis, especially when evaluating drug penetration and metabolism (104).

Most exciting, PDOs offer new possibilities as powerful preclinical models able to account for interpatient variability and offer the hope of personalized medicine. Conventional methods for growing organoids, similar to other 3D cell culture models, require a matrix basement. Organoids are generated from individual cells or fragments from primary tissues and then embedded in a matrix such as Matrigel, a basement membrane derived from Engelbreth–Holm–Swarm mouse sarcoma extracellular matrix forms a 3D gel (101, 105). The organoid is then propagated in the Matrigel until it has reached a sufficient size and/or acquired the phenotypic properties of its organ of interest, for example, the crypt and villi structure of the colon.

While Matrigel is essential to grow organoids, its molecular composition presents a significant complication in MSI analysis. The small molecules and peptides in Matrigel and other basement membrane substrates cause interfering signals and complicate MSI data analysis. In order to reduce this background contribution, Johnson *et al.* (106) developed a centrifugation method to optimize sample preparation steps for MSI experiments. By washing and microcentrifuging, organoids could be separated from Matrigel to remove the matrix and then centrifuged to a gelatin microarray. This technique reduces background interference and increases the throughput of organoids, allowing tens to hundreds of organoids analyzed in different sections without damaging the samples. The improvement makes the organoid preparation approach more compatible with the MSI method.

A huge potential clinical benefit of MSI on organoids is the potential to test patient-specific responses to a drug and to study the drug's biotransformation. Liu *et al.* established colon tumor organoids (CTOs) from two patients and successfully mapped irinotecan (*m/z* 587.3), IS irinotecan-d10 (*m/z* 597.3), and its metabolites, including a bioactive metabolite SN-38 (*m/z* 393.1) and an inactive metabolite SN-38 glucuronide (SN-38G) (*m/z* 569.1) using MALDI-MSI. By imaging irinotecan-treated and untreated CTOs at different time points, higher signal intensity was detected on the edge of organoids within 6 h, but the drug was more abundant in the center part of CTOs after 24 h of treatment, indicating full penetration. By comparing CTO samples treated with different drug concentrations, it was found that irinotecan uptake is a concentration-dependent process, with a higher drug concentration causing greater drug uptake and metabolism. This methodology could help better understand the difference of drug distribution and metabolism in different cell types from diverse aspects (107). Beyond exploring the effects of concentration, this methodology could also be powerfully applied to study of differential drug responses as a result of somatic mutations or epigenetics. The detection of metabolites from the therapeutic in a PDO could be a powerful predictor of individual clinical response.

**Figure 8:**
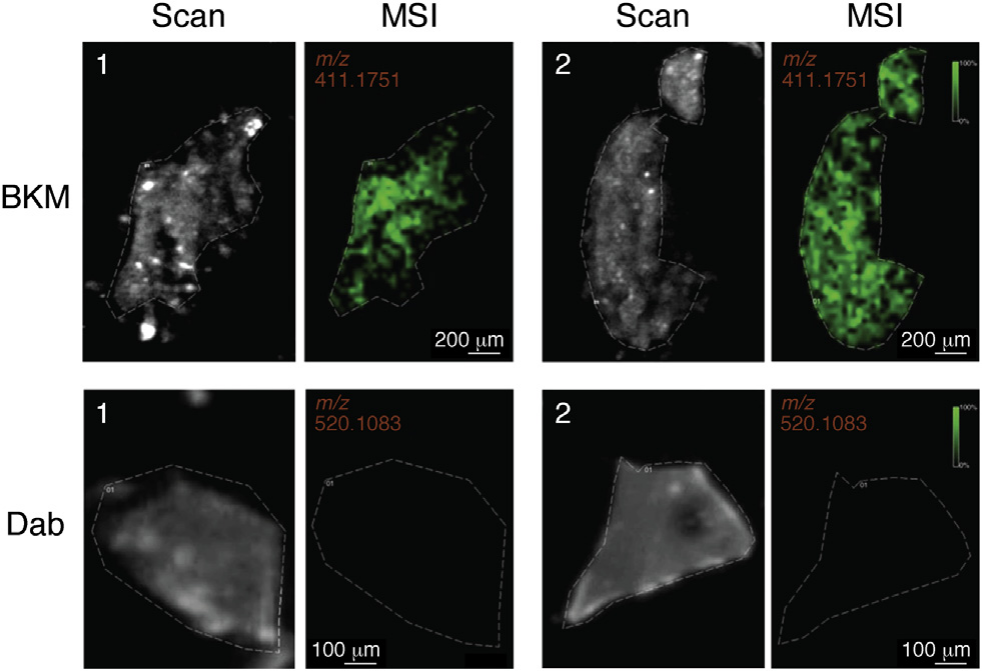
**Analysis of BKM120 and dabrafenib penetration by MALDI–MSI.** BBB organoids were incubated with 10 μM of either drug for 24 h. MALDI-MSI ion images show successful detection of BKM120 accumulation within the organoids (*green*; top row; *m/z* = 411.1751 ± 0.001). Dabrafenib was not detected within the organoids (*bottom row*; *m/z* = 520.1083 ± 0.001). *Dashed lines* on the scanned images delineate the positions of the BBB sphere tissue sections. This figure is adapted from Ref. (116) under a Creative Commons Attribution 4.0 license (https://creativecommons.org/licenses/by/4.0/legalcode). BBB, blood–brain barrier; MSI, MS imaging.

Organoids can be prepared from most soft epithelial lesions and have successfully been grown from pancreas (108), breast (109), and bladder cancers (110). Unlike traditional cell cultures, organoids can also be easily grown from cells in nonmalignant tissues. For example, Bergmann *et al.* demonstrated that multicellular blood–brain barrier (BBB) organoids can be used to evaluate brain-penetrating compounds. By coculturing endothelial cells, pericytes, and astrocytes, they created a standard protocol of making BBB organoids and used MALDI-cyclotron FTICR-MSI to monitor the distribution of two drugs within the organoids to compare their different permeability. BKM120, a phosphatidylinositol 3-kinase inhibitor known to penetrate BBB, was detected at high signal intensity (*m/z* 411.1751), whereas dabrafenib, a serine/threonine–protein kinase B–Raf inhibitor known not to cross BBB, was not detected in the images (*m/z* 520.1083). The results correspond with the known permeability of these two drugs and indicate that the BBB organoids have great ability in predicting whether the therapeutic compounds of interest could cross the BBB (111) (Fig. 8).

These studies show that organoids are reliable platforms for label-free MSI study. The employment of organoids advances the field beyond the limitations of spheroids, extending the potential to improve the preclinical assessment for *in vivo* therapeutics.

## Conclusions

Determining the distribution of drugs and their metabolites is critical in disease pharmacology. The developments of MSI have made it a powerful tool for personalized medicine and drug discovery, especially when combined with 3D cell culture model such as MCTS and organoids, which can better mimic the chemical microenvironment in patient tumors. Continued improvements in the MSI techniques, including ionization methods, sample preparation protocols, and data analysis approaches, maximize the information obtained in these studies.

Different ionization techniques have been developed for MSI, such as SIMS, DESI, and LAICP to provide more options and extend the application of MSI to map different molecules with high resolution; of these, MALDI remains a popular technique for MSI because of its large detection range and high sensitivity. By washing the sample slides, changing the matrix, or adding a derivatization reaction, detection of specific target molecules can be increased in imaging. In particular, the latest research has shown that MALDI-MSI has a great ability to monitor the distribution of various novel drugs in spheroids and organoids and the concomitant production of their metabolites. These spatial maps of both the drug and its resulting metabolites are powerful tools to better understand the drug mechanism and the response in metabolism pathways.

By continuing adapting MSI data analysis technologies to spheroids and organoids, especially the development of different open-source software and the application of ML to manage the large dataset of MSI, it has the potential to become a more informative and user-friendly platform to screen and select drugs for patients.

Quantitative studies have been completed using LAICP to accurately study the concentration of some metal-based drugs in spheroids, but the high cost and limited target molecules make quantification remain challenging. With continued improvements in MSI, the quantification imaging on spheroids and organoids could be optimized, enabling the rigorous quantitative analysis of individual spheroids and organoids.

Most exciting, the application of MSI technologies to PDOs offers the promise of personalized drug selection. As up to a dozen organoids can be prepared from a single patient biopsy, several compounds or combinations of compounds could be used to treat replicate organoids from the same patient. With MSI analysis, the molecular response of those organoids, including the mapping of their resulting metabolites, could be accomplished in a reasonable time window, thus providing critical information for clinicians and patients.

## Conflict of interest

The authors declare that they have no conflicts of interest with the contents of this article.
